# Menstrual Cycle Characteristics in Women With and Without Thyroid Disease

**DOI:** 10.7759/cureus.62724

**Published:** 2024-06-19

**Authors:** Gökçen Güngör Semiz, Zeliha Hekimsoy

**Affiliations:** 1 Endocrinology and Metabolism, Buca Seyfi Demirsoy Training and Research Hospital, Izmir, TUR; 2 Endocrinology and Metabolism, Celal Bayar University, Manisa, TUR

**Keywords:** thyroid dysfunction, irregular menstruation, menstruation disturbances, hypothyroidism, hyperthyroidism

## Abstract

Introduction: This study was designed to evaluate the frequency and type of menstrual disorders in thyroid dysfunction. The relationship between thyroid dysfunction and menstrual disorders has been known for a long time. The menstrual cycle should be checked in women with thyroid dysfunction. On the contrary, women with menstrual irregularities should be investigated for thyroid dysfunction.

Methods: Women who presented to our hospital’s internal medicine and endocrinology clinics that recently diagnosed thyroid dysfunction were included. The patients were divided into five groups (subclinical hypothyroidism, overt hypothyroidism, subclinical hyperthyroidism, overt hyperthyroidism, and euthyroid) according to thyroid functions. They were questioned regarding the amount, frequency, and duration of menstrual bleeding. The prevalence of menstrual disturbances, including secondary amenorrhea, hypomenorrhea, oligomenorrhea, hypermenorrhea, polymenorrhea, menorrhagia, metrorrhagia, and menometrorrhagia, was examined in 485 patients and 108 healthy controls.

Results: Hypermenorrhea was significantly more common in patients with overt hypothyroidism (33%) than in controls (6%) (p<0.05). The types and frequencies of menstrual disorders in patients with hyperthyroidism and those with normal thyroid function were not significantly different from those in controls.

Conclusion: Menstrual abnormalities frequently occur in women with thyroid dysfunction. Therefore, menstrual dysfunction should be considered when treating patients with thyroid abnormalities.

## Introduction

The hypothalamic-pituitary-thyroid axis and the hypothalamic-pituitary-gonadal axis are physiologically intertwined. The function of the thyroid is under the control of the hypothalamic-pituitary-thyroid axis. Thyroid dysfunction has a great impact on reproductive function before, during, and after pregnancy. The most common endocrine condition affecting women's reproduction is thyroid disorder [1].

The relationship between thyroid dysfunction and menstrual disorders has been known for a long time; both hyperthyroidism and hypothyroidism can lead to menstrual disorders. The most common menstrual disorder in hyperthyroidism is oligomenorrhea, followed by amenorrhea and polymenorrhea, whereas, in hypothyroidism, hypermenorrhea is most common [2-4].

A recent review investigated menstrual disorders observed in various endocrine diseases. In this review, irregular menstruation, heavy bleeding, oligomenorrhea, and amenorrhea were seen in hypothyroid women, while oligomenorrhea and amenorrhea were seen in hyperthyroid women [5].

Previous studies have included few patients and lacked controls. Additionally, many did not include patients with subclinical hyperthyroidism or subclinical hypothyroidism [2].

The aim of this study was to evaluate the frequency and type of menstrual disorders in women with hypothyroidism, hyperthyroidism, and normal thyroid function. Patients without any pathology on physical and laboratory examinations were classified as controls. Furthermore, unlike prior studies, we included patients with subclinical hypothyroidism and hyperthyroidism.

## Materials and methods

The protocol for this study was approved by the Ethics Committee of the Celal Bayar University Faculty of Medicine (02/06/2011-172). Women of reproductive age (17-52) who presented to our hospital’s internal medicine or endocrinology clinics between June 2011 and December 2013 for recently diagnosed thyroid dysfunction (who had not yet begun treatment) were approached for inclusion in the study. Pregnant, lactating, and peri- and postmenopausal women were excluded, as were those with primary amenorrhea; polycystic ovary syndrome; galactorrhea; breast or ovarian carcinoma; other genital tract pathologies; chronic kidney failure; diabetes mellitus; chronic liver disease; inherited or acquired hemorrhagic disease or any hypothalamic, pituitary, or adrenal disease; and those taking any chemotherapy, hormonal contraceptives, anticoagulants, or any undergoing treatment for infertility. Furthermore, women who were undergoing treatment for previously diagnosed thyroid dysfunction were excluded from the study.

After obtaining informed consent, the patients were asked about the amount, frequency, and duration of menstrual bleeding. Information was sought to characterize menstrual irregularities, such as secondary amenorrhea, hypomenorrhea, hypermenorrhea, oligomenorrhea, polymenorrhea, menorrhagia, metrorrhagia, and menometrorrhagia (Table 1) [4,6,7].

**Table 1:** Description of menstrual disorders

Menorrhagia	80 mL blood loss per cycle or menstruation or menstruation lasting longer than a week
Oligomenorrhea	Regular bleeding with an interval longer than 35 days
Polymenorrhea	Regular bleeding with an interval shorter than 21 days
Hypermenorrhea	Bleeding with regular intervals and normal periods but increased amount of bleeding
Hypomenorrhea	Bleeding with regular intervals and normal periods but decreased amount of bleeding
Metrorrhagia	Frequent bleeding at irregular intervals
Menometrorrhagia	Excessive uterine bleeding with irregular intervals
Secondary amenorrhea	No bleeding during a minimum of three normal cycles

Blood levels of free triiodothyronine (fT3), free thyroxine (fT4), and thyroid-stimulating hormone (TSH) were measured, and thyroid Doppler ultrasonography was performed on all patients. Thyroglobulin antibodies (anti-Tg), thyroid peroxidase antibodies (anti-TPO), TSH receptor antibody (TRAb), prolactin (PRL), follicle-stimulating hormone, luteinizing hormone and estradiol (E2) were measured, and thyroid scintigraphy was performed in patients with indications.

For data analysis, patients without any pathology on physical and laboratory examinations were classified as controls.

Patients were evaluated as euthyroid if the TSH, fT3, and fT4 were within the normal ranges; when TSH was high with fT3 and fT4 within the normal ranges, they were labeled as subclinical hypothyroidism. Overt hypothyroidism was diagnosed when TSH was high; fT3 and fT4 levels were below normal. Subclinical hyperthyroidism was diagnosed if TSH was low and fT3 and fT4 levels were within the normal ranges, and overt hyperthyroidism was diagnosed when TSH level was low and fT3 and fT4 levels were high.

Data from patients with hypothyroidism (subclinical and overt) were divided into three groups for analysis to determine the type and frequency of menstrual disorder within each group: those with serum TSH of 5-10 μIU/mL (mild hypothyroidism), serum TSH of 11-50 μIU/mL (moderate hypothyroidism), and serum TSH >50 μIU/mL (severe hypothyroidism). The mean TSH levels were calculated for patients with each type of thyroid disorder and were compared with those in the controls

Statistical analysis 

Statistical Package for the Social Sciences, version 19 was used in the evaluation of the data. Qualitative variables are reported as means ± standard deviations and quantitative variables as numbers and percentages. Data were analyzed using the chi-square test, one-way analysis of variance, Dunnet T3, Mann-Whitney U test, and Student’s t-test. P-values of <0.05 were used to denote statistical significance.

## Results

In this study, 593 women, 485 patients (with thyroid dysfunction) and 108 controls, were included. The patient and control group consisted of women of reproductive age. The age distribution was 18-52 years in the patient group and 18-43 years in the control group. The distribution of patients according to thyroid function was as follows: 17.9% (n=106) had subclinical hypothyroidism, 9.1% (n=54) had overt hypothyroidism, 9.3% (n=55) had subclinical hyperthyroidism, 8.4% (n=50) had overt hyperthyroidism, and 37.1% (n=220) were euthyroid (Hashimoto and nodular goiter).

The prevalence rates of secondary amenorrhea, hypomenorrhea, hypermenorrhea, oligomenorrhea, polymenorrhea, menorrhagia, metrorrhagia, and menometrorrhagia in the patients and controls are shown in Table 2.

**Table 2:** Type and frequency of menstrual disorders in controls and in patients with thyroid dysfunction Data are shown as n (%). *p=0.001; hypermenorrhea was significantly more common in patients with overt hypothyroidism than in controls *The chi-square test was used for categorical variables

	Controls, 108 (%)	Patients: 485 (%)				
		Hypothyroidism		Hyperthyroidism		Euthyroid, 220 (%)
		Overt, 54 (%)	Subclinical, 106 (%)	Overt, 50 (%)	Subclinical, 55 (%)	
TSH μIU/mL	1.73±0.91	44.16±34.79	8.19±2.86	0.04±0.07	0.18±0.10	2.33±2.01
Secondary amenorrhea	2 (1.9)	3 (5.6)	3 (2.8)	0 (0)	3 (5.5)	12 (5.5)
Hypomenorrhea	9 (8.3)	4 (7.4)	8 (7.5)	5 (10)	4 (7.3)	14 (6.4)
Hypermenorrhea	6 (6)	18 (33)*	20 (19)	8 (16)	11 (20)	34 (16)
Oligomenorrhea	13 (12)	14 (26)	29 (27)	10 (20)	10 (18)	49 (22)
Polymenorrhea	13 (12)	11 (20)	13 (12)	7 (14)	11 (20)	23 (10)
Menorrhagia	10 (9)	14 (26)	21 (20)	6 (12)	12 (22)	46 (21)
Metrorrhagia	2 (2)	3 (6)	7 (7)	2 (4)	3 (6)	10 (4)
Menometrorrhagia	0 (0)	2 (4)	4 (4)	0 (0)	1 (2)	4 (2)

Hypermenorrhea was significantly more common in patients with overt hypothyroidism (33%) than in controls (6%) (p=0.001). However, the prevalence rates of menorrhagia (26%), polymenorrhea (20%), and oligomenorrhea (26%) in patients with overt hypothyroidism and controls (9%, 12%, and 12%, respectively) were not significantly different.

Patients with subclinical hypothyroidism and overt hypothyroidism were divided into three groups (i.e., mild, moderate, and severe) according to serum TSH levels. When the group with TSH >50 μIU/mL was compared with the group with TSH 5-10 μIU/mL, a significant difference in the prevalence of hypermenorrhea was observed (p=0.022). Table 3 shows the relationship between the level of hypothyroidism and menstrual disorders.

**Table 3:** Type and frequency of menstrual disorders in patients with mild, moderate, and severe hypothyroidism Data are shown as n (%). *p=0.022 (when patients with mild and severe hypothyroidism were compared) *The chi-square test was used for categorical variables

	Mild TSH 5-10 µIU/mL, n (%)	Moderate TSH 11-50 µIU/mL, n (%)	Severe TSH >50 µIU/mL, n (%)
Secondary amenorrhea	3 (3)	0 (0)	3 (15)
Hypomenorrhea	7 (8)	2 (4)	3 (15)
Hypermenorrhea	15 (16)	16 (33)	7 (35)*
Oligomenorrhea	25 (28)	11 (22)	7 (35)
Polymenorrhea	11 (2)	8 (16)	5 (25)
Menorrhagia	18 (20)	10 (20)	7 (35)
Metrorrhagia	7 (8)	1 (2)	2 (10)
Menometrorrhagia	4 (4)	1 (2)	1 (5)

The types and frequencies of menstrual disorders in patients with hyperthyroidism and controls were not significantly different (p>0.05 in all comparisons). Oligomenorrhea was the most frequent disorder (20%) in overt hyperthyroidism, and its prevalence was not significantly different compared with that in the control group (p>0.05). Among patients with normal thyroid function (euthyroid Hashimoto and nodular goiter), oligomenorrhea was also the most frequent menstrual disorder, and its prevalence was not significantly different from controls (p>0.05).

## Discussion

Thyroid hormones influence most bodily systems, particularly reproductive functions. An association between thyroid dysfunction and menstrual disorders has been known for a long time; however, most studies included few patients and did not include a control group. Furthermore, the evaluation of patients in the subcategories of hypo- and hyperthyroidism was performed in a few studies.

Hypothyroidism alters the length of the menstrual cycle and the volume of bleeding in women of reproductive age. The latter is most likely brought on by anovulation-related estrogen breakthrough hemorrhage. Polymenorrhea and menorrhagia may also be caused by hemostasis factor deficiencies. The percentage of menstruation irregularity in hypothyroidism approaches 80% in the oldest published research. Menorrhagia and polymenorrhea were the most frequently reported disorders [2]. In more recent literature, the most common bleeding patterns in women presenting with abnormal uterine bleeding were oligomenorrhea (23%) and menorrhagia (21%). Hypothyroidism (22%) was more common than hyperthyroidism (6%) in women with abnormal bleeding [3]. 

In the 1999 study by Krassas et al., menstrual cycles were irregular in 40 (23.8%) of 171 women with hypothyroidism [8]. In their healthy controls, menstrual irregularity was found in 8.4%. In the study by Kakuno et al., involving 2052 women, menstrual irregularity was found in 15.8% of patients with hypothyroidism and 23.8% of the healthy controls [9]. In both studies, no statistically significant differences in the prevalence of menstrual irregularities were found between the patient and control groups. In our study, hypermenorrhea was significantly more common in patients with overt hypothyroidism (33%) than in controls (6%) (p<0.05). In this regard, it differs from Krassas’s and Kakuno's research [8,9]. In the study by Kakuno et al., the only statistically significant finding was that the incidence of menstrual irregularity was higher in those with TSH levels >100 μIU/mL (34.8%) than in those with TSH levels <100 μIU/mL (10.2%) [9].

In 2015, Urmi et al. reported on the effect of hypothyroidism on menstrual patterns and fertility in women in Bangladesh [10]. An abnormal menstrual cycle was experienced by 34% of women with hypothyroidism and 13.4% of women with normal thyroid function. It was statistically significant. The most common menstrual disorders in women with hypothyroidism were oligomenorrhea, polymenorrhea, and amenorrhea, respectively. Our study supports this study. However, the most common menstrual disorders in our patients were hypermenorrhea, oligomenorrhea, and menorrhagia, respectively.

Women with subclinical hypothyroidism were also included in our study. No difference in menstrual abnormalities was observed between women with subclinical hypothyroidism and the control group. These findings are in contrast to a recent study by Sebtain et al., who found a significantly higher incidence of oligomenorrhea in subclinical hypothyroidism [11].

In 2016, Ajmani et al. reported on the role of thyroid dysfunction in people with menstrual disorders [12]. Their study included 50 patients with menstrual disorders and 50 controls. While 44% of those with menstrual disorders had thyroid dysfunction, only 20% of the controls had thyroid dysfunction. Hypothyroidism was the most common abnormality found in 34% of patients. In a more recent study, thyroid dysfunction was found in 48% of women presenting with abnormal uterine bleeding, and hypothyroidism was found in most of them [13]. Our study supports the literature. The frequency of menorrhagia was significantly higher in patients with hypothyroidism.

Like hypothyroidism, hyperthyroidism also affects the menstrual cycle. In 2014, among patients with thyrotoxicosis examined by Krassas et al., 11% had hypomenorrhea, 2% had oligomenorrhea, and 7% had polymenorrhea, and the prevalence of any menstrual abnormality was not significantly different from that in controls [14]. Kakuno et al. also found no differences in the prevalence of menstrual abnormalities between the control and patient groups in their study involving 586 women with hyperthyroidism due to Graves’ disease. Our results showed a high prevalence of oligomenorrhea in women with hyperthyroidism. Consistent with the literature, the prevalence of these disorders was not significantly different from that observed in our control population.

In Kakuno’s study, the prevalence of secondary amenorrhea was significantly higher in cases of severe hyperthyroidism (fT3 >30 pg/mL) than in those with moderate hyperthyroidism (fT3 <30 pg/mL) [9]. In our study, no relationship was found between the severity of hyperthyroidism and the frequency of menstrual disorders. The difference in our findings may be because of the lower number of patients in our study (n=105) than that in Kakuno’s study (n=586).

Even in the setting of normal thyroid function, thyroid autoimmunity may significantly affect reproductive activity and pregnancy. In Kakuno’s study, 558 patients with euthyroid chronic thyroiditis (antithyroid antibody-positive) were compared with controls in terms of the prevalence of menstrual disorders. Oligomenorrhea was found in 6.1%, polymenorrhea in 2.5%, and secondary amenorrhea in 1.4%; these percentages were not significantly different from those observed in their control group [9]. In our study, in line with the literature, oligomenorrhea was the most common menstrual disorder (22.3%) in the euthyroid group, and this prevalence was not significantly different from that in the control group.

The strengths of our study include the inclusion of a control group, the examination of subclinical patient groups, and the questioning of patients' menstrual patterns by a single individual. The limitations of our study include the absence of evaluations of parameters affecting menstrual patterns, such as body mass index, and the duration of thyroid dysfunction, in both the patient and control groups.

## Conclusions

Menstrual abnormalities occur frequently in women with thyroid dysfunction. Those with thyroid abnormalities should be asked about menstrual dysfunction. Because abnormal thyroid function may have negative effects on pregnancy and fertility, making a diagnosis and beginning treatment early may be important for many of these women. Conversely, thyroid function should be evaluated in women presenting with abnormal uterine bleeding.
